# Supplementary material for: Probing Spatiotemporal Effects of Intertrack Recombination with a New Implementation of Simultaneous Multiple Tracks in TRAX-CHEM
Source: Int J Mol Sci. 2025 Jan 10;26(2):571. doi: 10.3390/ijms26020571 (PMC11765274; doi:10.3390/ijms26020571)
Supplement: Supplementary file 1 [file ijms-26-00571-s001.zip › ijms-3349417-supplementary.pdf]

Supplementary Materials: Probing intertrack effects at ultra-high dose rates with a new implementation of simultaneous multiple tracks in TRAX-CHEM

Lorenzo Castelli[1,2,3], Gianmarco Camazzola [4], Martina C. Fuss[5], Daria Boscolo[4], Michael Kraemer[4], Valentina Tozzini[3,6], Marco Durante[4], Emanuele Scifoni[2]

1. Statistical Fluctuations

Figure S1 illustrates the fluctuations of G-value at 1 µs. The number of independent simulation usually vary from a couple of hundred keV of deposited energy up to 2/3 MeV depending on the radiation quality. It can be observed that as the number of independent simulations increases, both the mean value and standard deviation remain stable, while the standard error decreases as expected up to a value that is below 1%.

2. H_3O^+ ad OH^- Histograms

The behavior of H_3O^+ is in agreement with what is observed for primary radiolytic products in Section 3.2, hydroxyl ions on the other side follow a trend similar to the one observed for $H^\bullet$ (it is important to notice that OH^- results are conditioned from low statistic for protons).

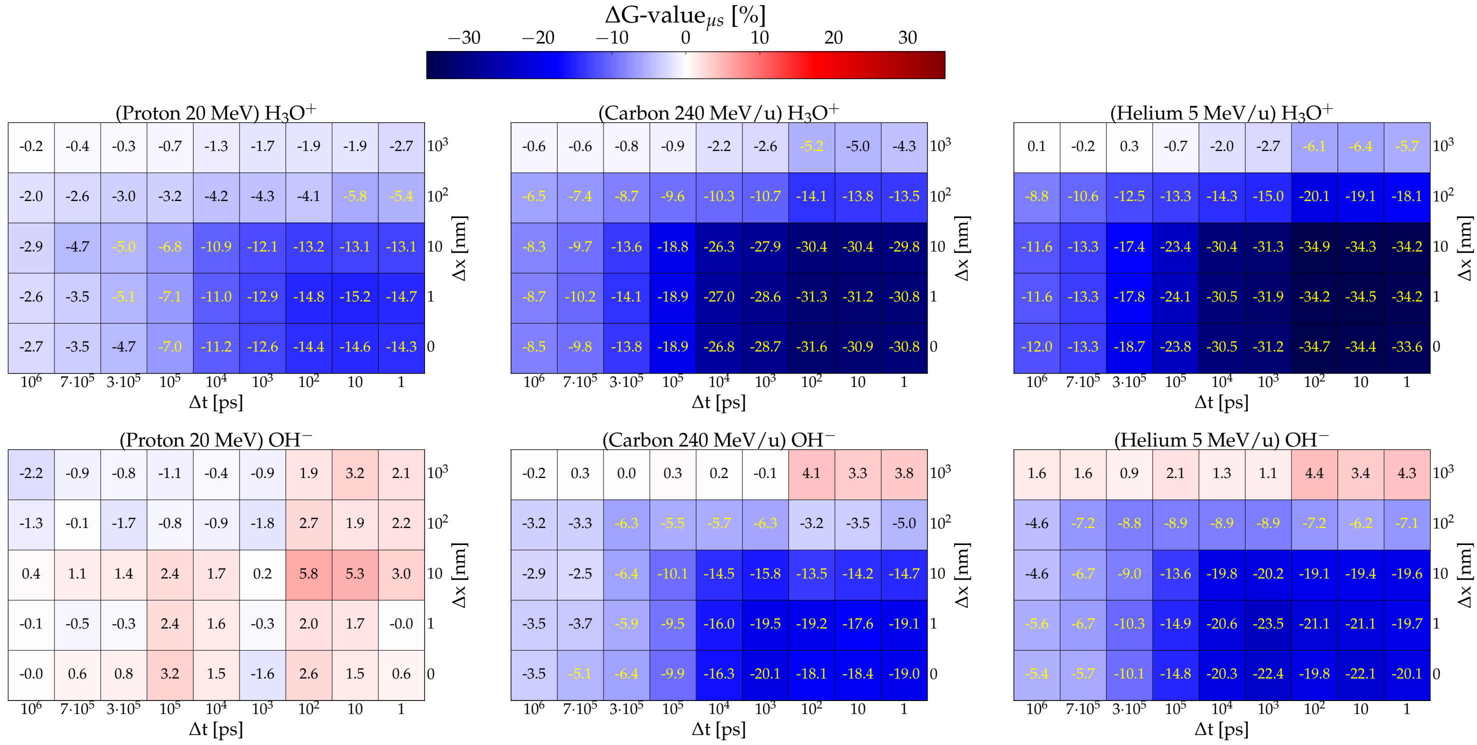

Figure S2. The ΔG-value$_{\mu s}$ for two chemical species—H_3O^+ and OH^-—resulting from the interaction of two 20 MeV protons (first column), 240 MeV/u carbon ions (second column), and 5 MeV/u helium ions (third column) in pure water, is shown as a function of the spatial separation Δx and temporal separation Δt between tracks. Each bin represents the relative difference between the intertrack effect and the NI scenario.

3. Proton and Helium comparison with PARTRAC

To compare the result with Kreipl's [1], the calculated G-values have been evaluated after 1 μs as a function of Δt and Δx, for 20 MeV Proton and 5 MeV/u Helium. Each column

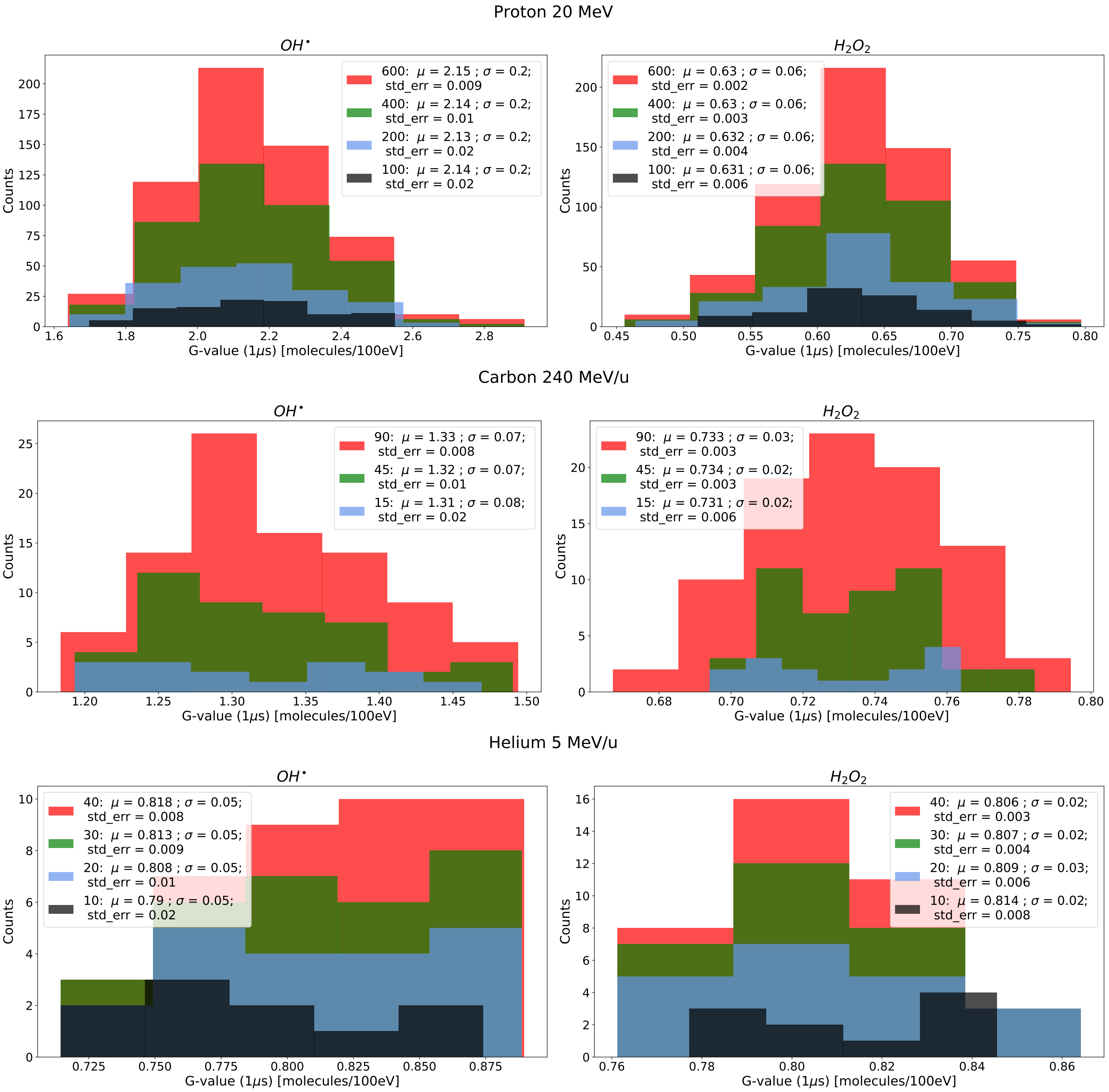

Figure S1. Histograms of the G-value at 1 µs and irradiation condition $\Delta t = 10$ ps and $\Delta x = 1000$ nm for Proton, Carbon, and Helium. The plots depict the histograms for $OH^{\bullet}$ and H_2O_2 across varying numbers of independent simulations, spanning released energies (in target volume) from approximately 500 keV to over 2.5 MeV. The legends provide the mean value (µ), standard deviation (σ), and standard error ($\sigma/\sqrt{N}$, N is the number of independent simulation).

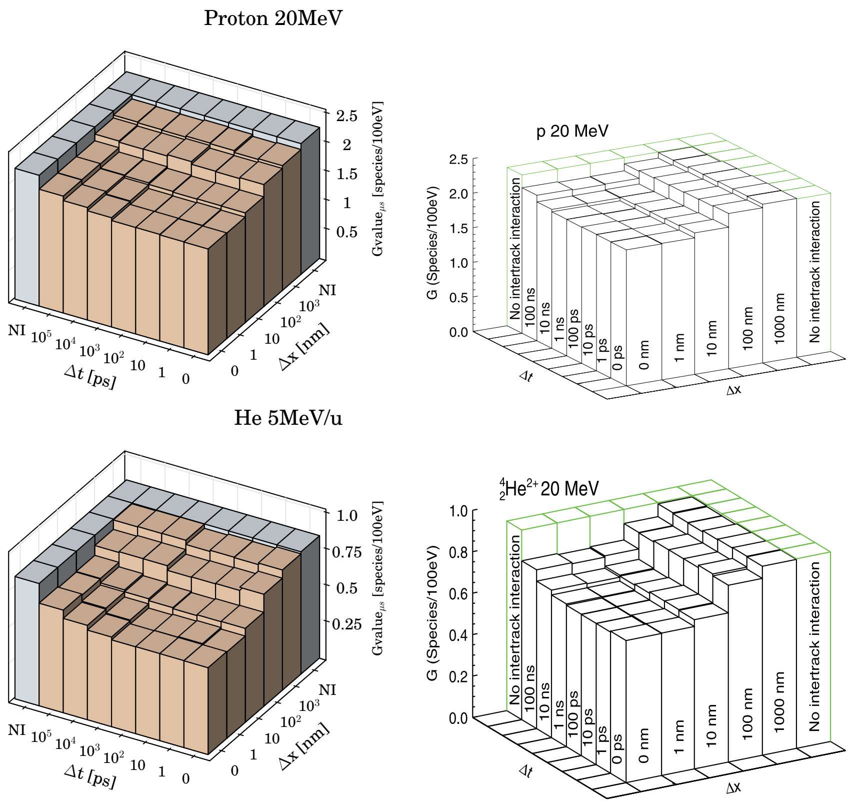

Figure S3. Calculated G-values of $OH^{\bullet}$ radicals at the end of the chemical stage (at 1µs) produced by a pair of the indicated ion type in pure water as a function of their spatial separation Δx and temporal separation Δt compared to the limit without intertrack interaction (gray bars on the left and green bars on the right). Left, the results obtained in this work, right, results obtained by Kreipl *et al.* [1].

in the three-dimensional plot in Figure S3 shows the G-value as a function of the temporal and spatial separation. These work results are displayed on the left side, and the gray row of columns at the rear and right side corresponds to the G-value for the limiting case NI. The green bars on the right side, from PARTRAC, also represent the same G-value data.

In PARTRAC simulations, G-values for radiolytic species like $OH^{\bullet}$ remain unaffected by intertrack reactions when the spatial separation between ion tracks exceeds 1 µm, consistent with TRAX-CHEM results for helium. However, for proton, TRAX shows a slight decrease in $OH^{\bullet}$ yields due to differing cross-sections, leading to broader spatial distributions of tracks. At Δx= 100 nm, both PARTRAC and TRAX-CHEM show slightly reduced radical yields across all ion types, with low dependency on Δt. At smaller separations (Δx= 10 nm), yields begin to depend considerably on Δt, with PARTRAC showing notable changes between 10 and 100 ns, while TRAX-CHEM indicates a gradual dependency starting from 100 ps. For $\Delta x < 10$ nm, yields drop further due to close track proximity, with no difference observed in either method.

4. ΔG-value for Carbon ions 40 MeV/u

By increasing the LET of Carbon ions to a level comparable to Helium, it becomes evident that the behavior of various chemical species remains consistent for the selected values. Of particular interest is the trend observed for H_2O_2, where a depletion appears—previously visible only with Helium and not with Carbon.

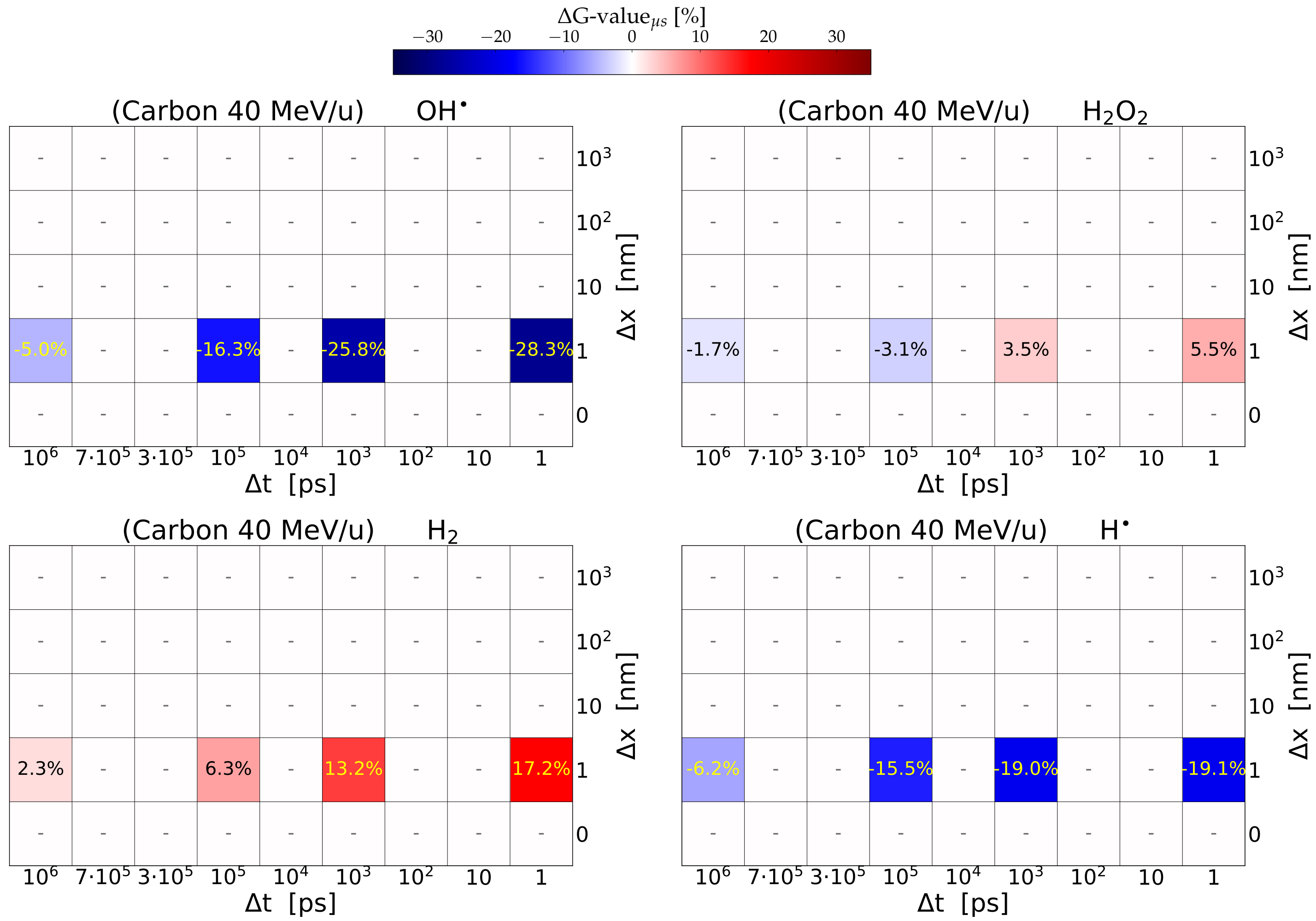

Figure S4. The ΔG-value$_{\mu s}$ for four chemical species—$OH^{\bullet}$ H_2O_2, H_2, and $H^{\bullet}$—resulting from the interaction of two 40 MeV/u carbon ions (LET $\sim$ 30 keV/µm) in pure water, is shown as a function of the spatial separation Δx and temporal separation Δt between tracks. Each bin represents the relative difference between the intertrack effect and the NI scenario. The values of the spacetime combinations other than $\Delta x = 1$ nm at $\Delta t = [1, 10^3, 10^5, 10^6]$ ps are not reported and are represented in the plot with "-".

1. Kreipl, M.; Paretzke, H. Interaction of ion tracks in spatial and temporal proximity. *Radiation and Environmental Biophysics* **2009**, *48(1)*, 349–359.

Disclaimer/Publisher's Note: The statements, opinions and data contained in all publications are solely those of the individual author(s) and contributor(s) and not of MDPI and/or the editor(s). MDPI and/or the editor(s) disclaim responsibility for any injury to people or property resulting from any ideas, methods, instructions or products referred to in the content.
